# Hydrogen Peroxide Detection by Super-Porous Hybrid CuO/Pt NP Platform: Improved Sensitivity and Selectivity

**DOI:** 10.3390/nano10102034

**Published:** 2020-10-15

**Authors:** Rakesh Kulkarni, Sundar Kunwar, Rutuja Mandavkar, Jae-Hun Jeong, Jihoon Lee

**Affiliations:** Department of Electronic Engineering, College of Electronics and Information, Kwangwoon University, Nowon-gu, Seoul 01897, Korea; rkulkarninanotech@gmail.com (R.K.); kunwarankees23@gmail.com (S.K.); rutuja.27rrm@gmail.com (R.M.)

**Keywords:** H_2_O_2_ detection, super-porous CuO/Pt electrode, dynamic hydrogen bubble technique, biosensor kit

## Abstract

A super-porous hybrid platform can offer significantly increased number of reaction sites for the analytes and thus can offer advantages in the biosensor applications. In this work, a significantly improved sensitivity and selectivity of hydrogen peroxide (H_2_O_2_) detection is demonstrated by a super-porous hybrid CuO/Pt nanoparticle (NP) platform on Si substrate as the first demonstration. The super-porous hybrid platform is fabricated by a physiochemical approach combining the physical vapor deposition of Pt NPs and electrochemical deposition of super-porous CuO structures by adopting a dynamic hydrogen bubble technique. Under an optimized condition, the hybrid CuO/Pt biosensor demonstrates a very high sensitivity of 2205 µA/mM·cm^2^ and a low limit of detection (LOD) of 140 nM with a wide detection range of H_2_O_2_. This is meaningfully improved performance as compared to the previously reported CuO-based H_2_O_2_ sensors as well as to the other metal oxide-based H_2_O_2_ sensors. The hybrid CuO/Pt platform exhibits an excellent selectivity against other interfering molecules such as glucose, fructose, dopamine, sodium chloride and ascorbic acid. Due to the synergetic effect of highly porous CuO structures and underlying Pt NPs, the CuO/Pt architecture offers extremely abundant active sites for the H_2_O_2_ reduction and electron transfer pathways.

## 1. Introduction

Hydrogen peroxide (H_2_O_2_) is one of the most important elements in the field of biomedical, environmental analysis, textile and food manufacturing industries due to its strong oxidizing property [1]. Also, it plays a crucial role as a signaling molecule in regulating various biological processes [2]. Furthermore, H_2_O_2_ has emerged as a key byproduct for many enzymatic reactions for the biosensing and diverse commercial industries [2]. The significant importance of H_2_O_2_ detection in various fields attracts many research groups to develop efficient sensors. Here, what is required is high sensitivity with a low limit of detection (LOD) along with good selectivity and a fast response.

**Figure 1:**
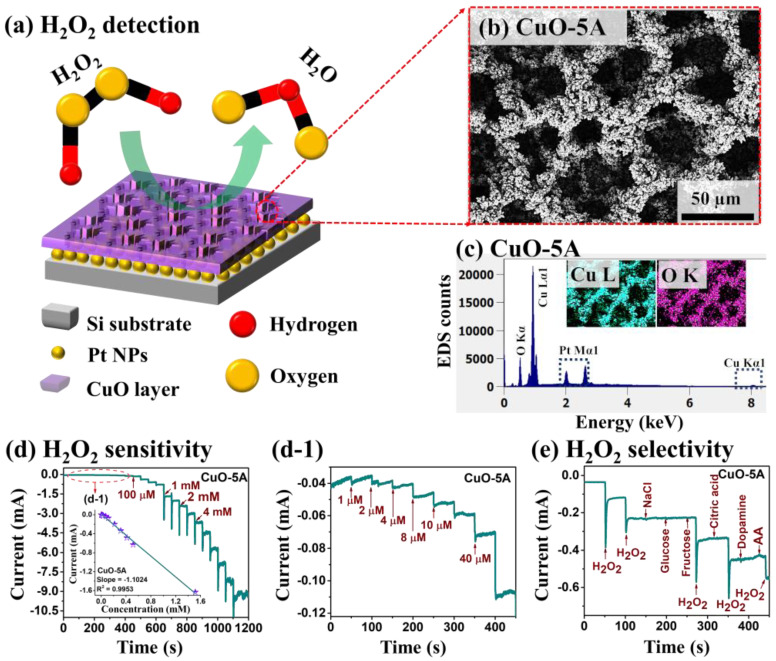
(**a**) Schematic representation of H_2_O_2_ detection by super-porous hybrid platform, made of CuO nanostructures on Pt nanoparticles (NPs) (CuO/Pt). (**b**) Scanning electron microscope (SEM) image of typical porous CuO nanostructures. (**c**) Corresponding energy-dispersive X-ray spectroscopy (EDS) spectra and elemental maps. (**d**,**d-1**) Amperometric response of CuO-5A sample upon the dropwise addition of H_2_O_2_ concentration from 1 µM–4 mM in a 0.1 M phosphate-buffered saline (PBS) solution of a pH 7.4 at −0.4 V potential. (**e**) Selectivity response of CuO-5A upon the successive addition of 0.1 mM H_2_O_2_, NaCl, glucose, fructose, citric acid, dopamine and ascorbic acid (AA) to 0.1 M PBS (pH 7.4).

Various techniques have been developed for the H_2_O_2_ detection including the colorimetric assay [3,4], fluorescence detection [5,6], electrochemical luminescence [7,8], surface-enhanced Raman spectroscopy (SERS) [9,10] etc. Among these, electrochemical sensing [11] can offer one of the handiest approaches for the detection of H_2_O_2_ due to its high sensitivity, fast response, precision and simple operation. The working principle of electrochemical H_2_O_2_ detection is based on the reduction of H_2_O_2_ into H_2_O by the active electrode materials [12]. The sensitivity, selectivity and LOD of H_2_O_2_ sensing directly depend upon the morphological, electrical and catalytic properties of the sensing electrodes [13]. In terms of the electrode materials, recently, noble metallic nanoparticles (NPs) have gained much attention in H_2_O_2_ detection due to the small particle size, high surface area and high electrocatalytic activity [14,15,16]. At the same time, metal oxides such as CuO, NiO, MnO_2_ and Ag_2_O nanostructures have emerged as a new class of materials for non-enzymatic electrochemical sensors [17,18,19,20,21]. Among various metal oxides, the CuO as a p-type semiconductor having 1.2 eV bandgap can offer high stability, better electrochemical properties and low manufacturing cost [19,20,21]. On the other hand, the Pt NPs are well known for their excellent catalytic activity and higher stability over other metals as well as high electrical conductivity [15,21]. Thus, the combination of CuO structures such as highly porous CuO nanostructures on a Pt NP template could provide a significantly increased number of active sites and improved catalytic activity for the H_2_O_2_ reduction and efficient electron transfer pathways for the electrochemical detection. The fabrication of highly porous CuO nanostructures is enabled by the dynamic hydrogen bubble technique. This platform including the porous CuO nanostructures and Pt NPs on Si substrate has been demonstrated for the first time in this work. Often, the CuO layers were utilized for the H_2_O_2_ detection but the porous nanostructures of CuO by the dynamic hydrogen bubble technique has been demonstrated first time in this work. Figure 1c shows the energy-dispersive X-ray spectroscopy (EDS) spectra of CuO-5A sample with the corresponding maps. The super-porous CuO/Pt hybrid platform demonstrates a high sensitivity of 2205 µA/mM·cm^2^ for the H_2_O_2_ detection as seen in Figure 1d and an excellent selectivity against glucose, fructose, dopamine, sodium chloride, citric acid and ascorbic acid as clearly seen in Figure 1e. It also shows a low LOD of 140 nM with a wide detection range. This is the first demonstration of super-porous CuO nanostructures and of the hybrid architecture with the Pt NPs for the H_2_O_2_ sensing.

In this work, a novel H_2_O_2_ sensing platform is demonstrated by the super-porous hybrid CuO nanostructures on Pt NPs, i.e., CuO/Pt, on Si substrate. The schematic representation of H_2_O_2_ molecule detection is represented in Figure 1a. The super-porous CuO/Pt hybrid electrode is demonstrated by a physical vapor deposition of Pt NPs on Si substrate followed by the electrochemical deposition of porous CuO nanostructures as shown in Figure 1b.

## 2. Materials and Methods

### 2.1. Materials

Copper sulfate (CuSO_4_), sulfuric acid (H_2_SO_4_), glucose (C_6_H_12_O_6_), fructose (C_6_H_12_O_6_), dopamine (C_8_H_11_NO_2_), ascorbic acid (AA, C_6_H_8_O_6_), sodium chloride (NaCl), hydrogen peroxide (H_2_O_2_), citric acid (C_6_H_8_O_7_) and phosphate-buffered saline tablets (PBS) were purchased from Sigma–Aldrich (St. Louis, Mo, United States). All the reagents were of analytical grade and used without further purification. Deionized (DI) water was used as a solvent throughout the experiment.

### 2.2. Fabrication of Pt Nanoparticles (NPs)

Initially, the Si substrate was degassed in the pulsed laser deposition (PLD) (DaDa TG, Daegu, South Korea) chamber under the 1.0 × 10^−4^ Torr at 725 °C for 30 min to remove the trapped gases, water vapors and contaminants. After degassing, the substrate was transferred to a plasma-assisted sputtering chamber for the deposition of 50 nm Pt film with the ionization current of 7 mA under 1.0 × 10^−1^ Torr. Subsequently, the Pt deposited Si sample was annealed in the PLD chamber at 425 °C for 30 min to form the Pt NPs and enhance the adhesion of Pt on Si. During the annealing process, the chamber pressure was kept constant at 1.0 × 10^−4^ Torr and the temperature was increased at 4 °C/s to reach the target temperature. To finish the sample growth, the heating system was turned off and the sample was kept under the same vacuum until the temperature was dropped to an ambient over time. The surface morphology of the Pt/Si sample after the annealing process is shown in Figure S1, which shows the root mean squared (RMS) roughness (Rq) and surface area ratio (SAR) were much increased with the formation of Pt NPs.

### 2.3. Fabrication of CuO Nanostructures

The electrochemical deposition of Cu was carried out in a three-electrode system comprising the Pt/Si substrate, platinum (Pt) electrode and Ag/AgCl electrode, as working (WE), counter (CE) and reference (RE) electrodes, respectively. The working electrode size was 0.5 × 1 cm^2^. For the deposition of porous Cu film on the Pt/Si, the precursor solution of 0.1 M CuSO_4_ and 0.05 M H_2_SO_4_ was prepared in 20 mL DI water [22]. Then, various Cu layers were deposited by varying the deposition time such as 5, 10, 20, 30 and 50 s at a fixed current density of 2 A/cm^2^ and also at the deposition current density of 0.5, 1, 3 and 5 A/cm^2^ with the applied potential of 1 V (vs Ag/AgCl). Then, the samples were transferred to the PLD chamber for the oxidation of Cu. A stepwise annealing at 300 and 500 °C for 30 min each was equally adapted with the continuous O_2_ (20 CC) flow. The PLD chamber vacuum was 1.7 × 10^−1^ Torr during the annealing under the O_2_ supply. After the completion of the annealing process, the pure Cu metal layer was converted into the CuO through the oxidation [23]. The CuO samples are named as CuO-10 s, CuO-20 s, CuO-30 s, CuO-50 s and CuO-0.5A, CuO-1A, CuO-2A, CuO-3A and CuO-5A, respectively, based on the variation of deposition time and current density.

### 2.4. Physical Characterizations

The surface morphology of prepared CuO/Pt/Si samples was characterized by a scanning electron microscope (SEM, COXEM CX-200, Daejeon, Korea) and atomic force microscopy (AFM, Park Systems Corp. XE-70, Gyeonggi-do, South Korea). For an elemental characterization of samples, an energy-dispersive X-ray spectroscope (EDS, Noran System 7, Thermo Fisher, Waltham, MA, USA) was used under the spectral and imaging modes. For the Raman measurement, a NOST system (Nostoptiks, Gyeonggi-do, Korea) was utilized, which is integrated with the 532 nm laser, spectrograph (ANDOR sr-500), charge-coupled device (CCD) and various optics. All the electrochemical measurements were carried out with the Wizmac-1200Premium system (Wizmac, Daejeon, Korea).

### 2.5. Electrochemical Measurement

All electrochemical performances of as-prepared electrodes were measured on the Wizmac-1200Premium system (Wizmac, Daejeon, Korea). The as-prepared hybrid electrode was used as the working electrode, and the Ag/AgCl and Pt plate were used as reference and counter electrodes respectively. The working electrode, the CuO/Pt/Si sample, size was 0.5 × 1 cm^2^. 0.1 M PBS (pH~7.4) was used as electrolytes for H_2_O_2_ detection, and a specified concentration of H_2_O_2_ was added continuously to 0.1 M PBS under a stirring condition. In order to maintain the O_2_-free environment, N_2_ was purged into the electrolyte solution before the electrochemical measurements. All sensing performances were examined at ambient conditions.

## 3. Results and Discussion

**Figure 2:**
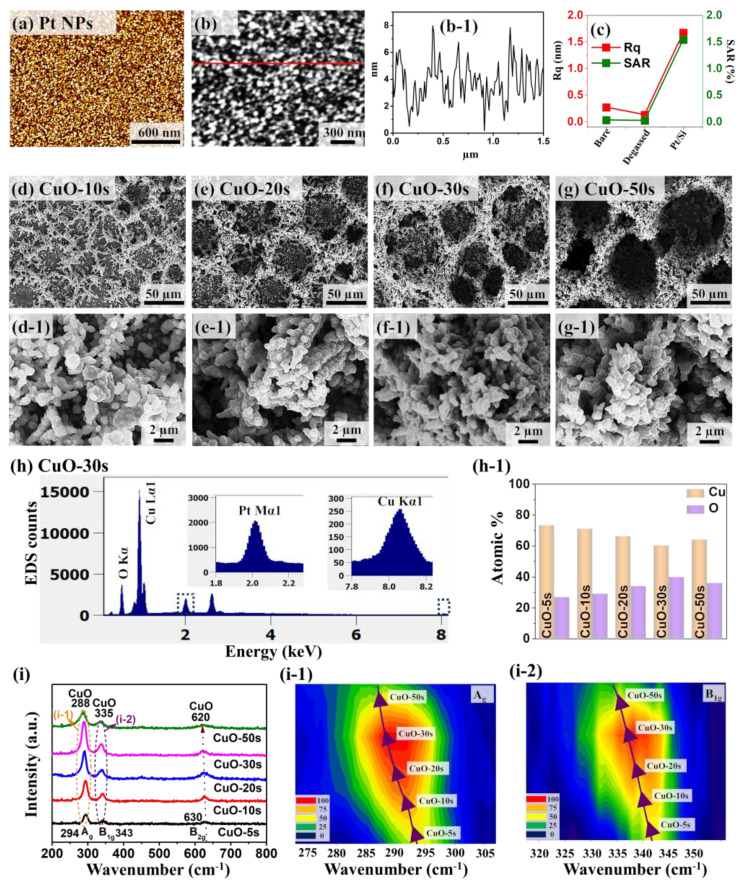
Structural analysis of porous CuO/Pt hybrid detectors fabricated at different deposition duration by an electrochemical deposition at 2 A/cm^2^ cathodic current density in a solution of 0.1 M CuSO_4_ and 0.05 M H_2_SO_4_. (**a**) Atomic force microscopy (AFM) top-view of Pt NPs on Si substrate. (**b**,**b-1**) Magnified AFM top-view and corresponding cross-sectional line-profile. (**c**) Rq and surface area ratio (SAR) plots at different conditions. (**d**–**g**) SEM images of the porous CuO/Pt hybrid nanostructures for different deposition durations from 10 to 50 s. The CuO-10 s stands for the ten-second deposition duration. (**d-1**–**g-1**) Corresponding zoom-in SEM images. (**h**) EDS spectra of CuO-30 s. (**h-1**) Summary of atomic percentage of Cu and O from different samples as a function of deposition time. (**i**) Raman spectra of the porous CuO/Pt hybrid samples. (**i-1**,**i-2**) Contour plots of the Raman peaks of CuO corresponding to the A_g_ and B1_g_ modes.

Figure 2 shows the physical properties of super-porous CuO/Pt hybrid electrodes by the variation of deposition-duration during the electrochemical deposition of Cu at 2 A/cm^2^ in a solution containing 0.1 M CuSO_4_ and 0.05 M H_2_SO_4_. Initially, the Pt nanoparticle (NP) templates were fabricated by the sputtering of 50 nm Pt film on Si substrate and subsequent annealing. The tiny Pt NPs were fabricated after annealing as clearly seen in the AFM top-views and line-profile in Figure 2a,b-1. Indeed, the Rq and SAR were significantly increased as shown in Figure 2c after the formation of Pt NPs. The average height and diameter of Pt NPs were estimated to be around 5 and 40 nm from the cross-sectional line-profiles in Figure 2b-1. With the annealing of Pt layers under the high temperature and vacuum, the strong adhesion between Pt and Si can be achieved as well, which could further facilitate the adsorption of Cu atoms during the electrochemical deposition. Figure 2d–g show the SEM images of highly porous CuO. The zoom-in SEM images are shown in Figure 2d-1–g-1. All the samples fabricated in this work clearly depicted the formation of highly porous structures as displayed the SEM images. Generally, the CuO nanostructures exhibited large surface pores as well as numerous small pores on the vertical side walls. Thus, the term “super-porous” is used to indicate such porous structures, which is achieved by the electrodeposition of Cu film along with the hydrogen bubbling. It can be observed that the partially connected CuO dendrites were formed on the surface of the Pt NP template with the deposition time (T_d_) of 10s in Figure 2d. As the T_d_ was increased, the gradual growth of interconnected porous structures was observed due to the additional deposition of Cu atoms in Figure 2e–g. The porous nature of CuO nanostructures was due to the simultaneous deposition of Cu and generation of hydrogen bubbles as described in Figure S2 [22]. Due to the large overpotential, the co-reduction process occurs, in which the Cu ions are reduced simultaneously with the H^+^ as described by Equations (1) and (2) [18].

(1) $$2H_{\left(\mathrm{aq}\right)}^{+}+\;2e^{-}\to {\;H}_{2}\;\;\;$$

(2) $${\mathrm{Cu}}_{\left(\mathrm{aq}\right)}^{2+}+\;2e^{-}\to {\;\mathrm{Cu}}_{s}$$

The generation of hydrogen bubbles functions as a dynamic bubble template for the porous Cu deposition. The pore size was found to be increased with the deposition time due to the coalescence of Cu nanostructures. Meanwhile, the intensive dendrites and corn-like agglomerates were grown towards the interior of the pores, resulting in the formation of highly porous 3D Cu nanostructures. The complete mechanism of CuO fabrication on Pt/Si substrate is shown in Figure S2 and additional SEM images are provided in Figure S3. The Cu particle size was not significantly affected as seen in Figure 2d-1–g-1.

After the fabrication of highly porous Cu nanostructures, each sample was annealed at 500 °C for 30 min under the continuous O_2_ (20 CC) flow, which converts the Cu into CuO by the oxidation without much difference in the morphology. Furthermore, the elemental characterization was carried out as shown in Figure 2h,h-1. The EDS spectra of other samples are provided in Figure S4. The EDS spectra reveal the presence of Cu, O and Pt elemental peaks, indicating the formation of CuO on the Pt NP template. In addition, the corresponding atomic percentage of Cu and O for each sample is summarized in Figure 2h-1. This indicates that the oxygen amount was gradually increased up to 30 s of Cu deposition and the oxygen amount was decreased at 50 s likely due to the deposition of a thicker Cu layer, which can prevent the exposure of Cu atoms during the annealing under O_2_. From the EDS analysis, it was also observed that the atomic percentage ratio of Cu to O is ~2:1. This could be due to the unreacted or unoxidized Cu deep in the structures. In addition, Raman scattering spectra were obtained from the CuO samples for the crystal phase characterization of the deposited materials as shown in Figure 2i. All samples exhibited three Raman peaks at 294, 343 and 630 cm^−1^, corresponding to the A_g_, B_1g_ and B_2g_ phonon modes of CuO [24]. The Cu_2_O phase showed the Raman peaks around 220, 415, 520 and 630 cm^−1^. This clearly indicates that the oxidation status of our samples is mostly CuO with the strong Raman peaks at 294 and 343 cm^−1^. It was observed that the intensity of Raman peaks was gradually increased up to the CuO-30 s, which may be due to the gradually increased size. It can also be observed from the previous results that the Raman peak position can slightly vary depending upon the annealing temperature and crystallinity of the samples [25]. However, with the 50 s, a lower intensity of Raman peak was observed, which could be due to the poor crystallinity by an inefficient oxidation with the thick structure formation as discussed with the EDS spectra [26]. The counterplots of A_g_ and B_1g_ peaks are shown in Figure 2i-1,i-2, which demonstrate the blue shift of the A_g_ and B_1g_ Raman peaks and broadening. The gradual peak broadening and shift in the Raman peaks at ~288, 335 and 620 cm^−1^ can be due to the gradually increased size effect [27,28]. When the deposition time was increased, the grain size was gradually increased, and the formation of thick CuO walls and formation of large dendrite structures were observed.

**Figure 3:**
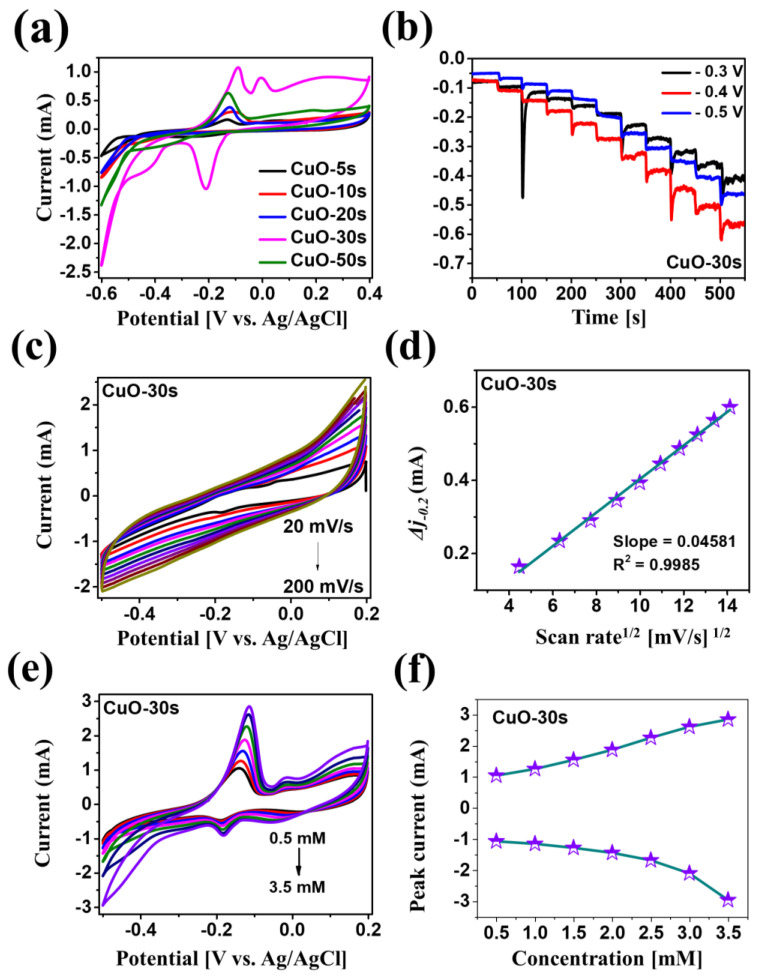
(**a**) Cyclic voltammetry (CV) response of various porous CuO/Pt hybrid detectors in 0.1 M PBS (pH 7.4) containing 0.4 mM H_2_O_2_ at a scan rate of 50 mV/s. (**b**) Amperometric response of CuO-30 s sample with the dropwise addition of 0.1 mM H_2_O_2_ at different applied potential. (**c**) CVs response of the CuO-30 s sample at different scan rates from 20 to 200 mV/s in 0.1 M PBS (pH 7.4) containing 0.1 mM H_2_O_2_. (**d**) Capacitive current Vs square root of scan rate calibration plots for the CuO-30 s at –0.2 V (*Δj*_−0.2_ = (*j_a_* − *j**_c_***)/2). (**e**) CV of CuO-30 s sample in 0.1 M PBS (pH 7.4) containing different concentrations of H_2_O_2_ ranging from 0.5 to 3.5 mM at the scan rate of 50 mV/s. (**f**) Relation between peak current with respect to H_2_O_2_ concentration at −0.5 V.

Figure 3 shows the electrochemical characterizations of the deposition-duration variation set via cyclic voltammetry (CV) and chronoamperometry (CA). The CV and amperometric response of Si and fabricated Si/Pt substrate with the addition of 0.1 M H_2_O_2_ in 0.1 mM PBS (pH 7.4) electrolyte is shown in Figure S5. First, the CV plots of CuO/Pt hybrid electrodes was measured in a stirred 0.1 M PBS (pH 7.4) containing 0.4 mM H_2_O_2_ at a scan rate of 50 mV/s as shown in Figure 3a. From the CV results, all the CuO samples showed a gradual increase in the oxidation and reduction peaks along with the deposition time up to 30 s. With the increase in the deposition time, the CuO thickness was increased in both vertical and lateral directions, which resulted in the evolution of porous CuO nanostructures. Due to the much-increased thickness of Cu for the CuO-50 s, the conversion rate of Cu to CuO was diminished, resulting in the increased atomic percentage of Cu as clearly demonstrated by the EDS and Raman spectra analyses in the previous section. The highly porous nature of the CuO-30 s is not only effective for the electron pathways but also provides a significantly increased number of active sites, which is helpful for the enhancement of electrochemical detection of H_2_O_2_. The overall mechanism of H_2_O_2_ reduction can be expressed by the relation [29]:

(3) $$H_{2}O_{2}+2\mathrm{CuO}\;\to {\;\mathrm{Cu}}_{2}O+{\;O}_{2}+{\;H}_{2}O$$

The electrocatalytic reduction of H_2_O_2_ by CuO can be described in two steps: (i) electrochemical reduction of Cu(II) to Cu(I) and (ii) electron transmission and O_2_ generation, reducing the H_2_O_2_ into H_2_O. From the CV measurement, the CuO-30 s showed the two high intensity reduction peaks at around −0.2 and −0.4 V and two oxidation peaks at −0.1 and 0 V in Figure 3a. The two reduction peaks can be corresponded to the stepwise one-electron reduction of Cu(II) to Cu(I) and Cu(I) to Cu(0), whereas the two oxidation peaks can likely correspond to the one-electron oxidation of Cu(0) to Cu(I) and of Cu(I) to Cu(II) [30]. Since the CuO-30 s demonstrated the highest oxidation and reduction peaks, it was further studied for the amperometric response at different applied potentials upon the drop-wise addition of 0.1 mM H_2_O_2_ solution as shown in Figure 3b. The maximum and stable current response was obtained at −0.4 V. It is well known that the applied potential in the CA has a great influence on the sensitivity, stability and selectivity of the sensor [31]. Figure 3c displays the CV response of the CuO-30 s by varying the scan rate in the range of 20–200 mV/s in a 0.1 M PBS (pH 7.4) containing 0.1 mM H_2_O_2_. With the higher applied scan rate, the peak potential was increased, which consequently indicates that the electrocatalytic activities are enhanced by the absorbed analytes at a higher scan rate [32]. The corresponding graph in Figure 3d demonstrates the linear plot of capacitive current (*Δj*_−0.2_) versus the square root of the scan rate, in which the slope of the plot corresponds to the double layer capacitance (C_dl_) of the electrode [33]. The C_dl_ can be utilized to qualitatively evaluate the electrochemical-active surface area (ECSA) of an electrode. Obviously, the higher C_dl_ can indicate the increased ECSA when the geometrical area is fixed. Here, the C_dl_ for the CuO-30 s sample was 0.04581 mF, which is a decently good value as compared with other conventional electrodes. Furthermore, the current versus potential relationship with the variation of H_2_O_2_ concentration was studied at the scan rate of 50 mV/s as shown in Figure 3e. With the increased concentration of H_2_O_2_ from 0.5 to 3.5 mM, the current intensities of reduction and oxidation peaks were gradually increased due to the strong electrolyte reaction of H_2_O_2_. The increased reduction current can be ascribed to the increased Cu(II) species from Cu(I) by means of simultaneous reduction of H_2_O_2_ [18]. Figure 3f shows the relation between the peak current at −0.5 V with respect to the H_2_O_2_ concentration. As the peak current increases progressively at −0.5 V, it can indicate the high electrochemical activity of H_2_O_2_ reduction around this voltage.

**Figure 4:**
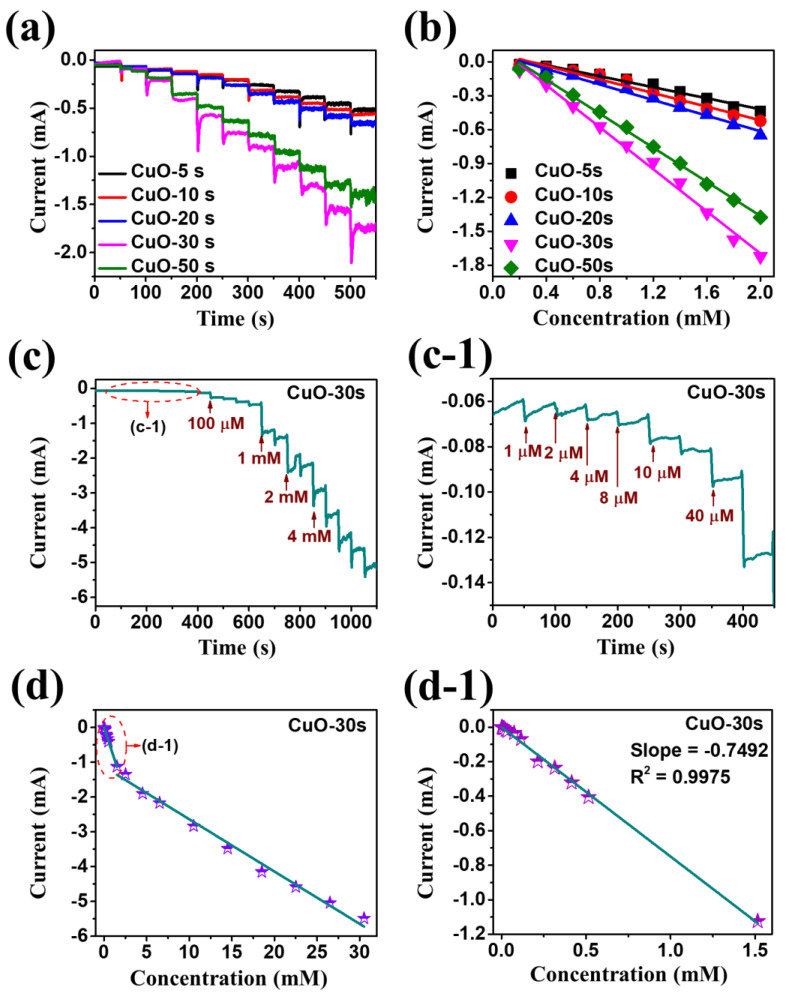
(**a**) Steady-state current time response of porous CuO/Pt hybrid detectors fabricated at a different duration between 5 and 50 s upon the successive addition of 0.2 mM H_2_O_2_ in N_2_- saturated 0.1 M PBS (pH 7.4) at an applied potential of −0.4 V. (**b**) Corresponding calibration curves of various CuO samples for H_2_O_2_ detection. (**c**,**c-1**) Amperometric response of CuO-30 s to dropwise addition of H_2_O_2_ from low (1 µM) to high (4 mM) concentrations. (**d**,**d-1**) Linear calibration curve of CuO-30 s based on the current versus H_2_O_2_ concentration at high concentration range from 0.1–4 mM and low concentration range from 1 µM–1.5 mM.

Figure 4 presents the cyclic voltammetry (CV) response for the electrochemical detection of H_2_O_2_ by the CuO samples fabricated at different deposition time. The CA response was measured by a dropwise addition of H_2_O_2_ concentrations in the N_2_-saturated 0.1 M PBS (pH 7.4) under a stirring condition at a potential of −0.4 V. The electrochemical CA response was performed using a three-electrode system comprising the Pt/Si substrate, platinum (Pt) electrode and Ag/AgCl electrode, as working (WE), counter (CE) and reference (RE) electrodes, respectively. Figure 4a shows the steady-state amperometric current response for the deposition time variation set upon the successive addition of 0.2 mM H_2_O_2_. The reduction current was sharply increased and stabilized approximately within 2 s after the ingestion of H_2_O_2_ drop. Specifically, the CuO-30 s demonstrated the highest current with a rapid electrochemical response among all samples. This can be due to the large active surface area of porous CuO nanostructures that allows the diffusion of H_2_O_2_ molecules with the best crystalline quality and efficient electron transfer at the interface during the reduction process. Figure 4b presents the current versus concentration relationship, in which the linear current response was observed as a function of concentration for all the samples and the CuO-30 s exhibited the highest current difference. Moreover, various concentrations of H_2_O_2_ were detected by the CuO-30 s as displayed in Figure 4c, which demonstrates that the current response was sharply increased upon the addition of each drop of H_2_O_2_ from 1 µM to 4 mM. To gain a clear understanding of low concentration H_2_O_2_ detection, the concentration regime from 1 µM–1.5 mM is separately plotted in Figure 4c-1, which displays a stepwise steady-state current after the successive addition of H_2_O_2_. In addition, Figure 4d shows the two linear region calibration curves for the low- and high-concentration ranges. The two linear regions can be observed due to the different activation and adsorption behavior of the hybrid CuO/Pt biosensor along with the increased H_2_O_2_ concentration [34]. Figure 4d-1 displays the linear regression at low concentration where the electrocatalytic mechanism is dominant, which is described by Equation (4) [9].

(4) $$y=ax\;+\;b\left(R^{2}\right)$$

*y* = −0.7492*x* − 6.4111 × 10^−4^ (R^2^ = 0.9976) for the first linear range. As the geometric surface area exposed to the electrolyte is 0.5 × 1 cm^2^, the sensitivity (S) is determined to be 1498 µA/mM·cm^2^. The definition of sensitivity (S) is shown in the Equation (5). The LOD is calculated to be 325 nM at a signal to noise (S/N) ratio of 3 based on the Equation (6) for the H_2_O_2_, where the σ represents the standard deviation [35]. Here, the *σ* was obtained to be 0.08114 µA by averaging 25 blank readings of CuO-30 s in the 0.1 M PBS electrolyte. Each reading was taken for 25 s.

(5) $$S=\frac{Slope}{Geometicla\;area\;of\;working\;electorde}$$

(6) $$LOD=\frac{3\times \sigma }{slope}$$

**Figure 5:**
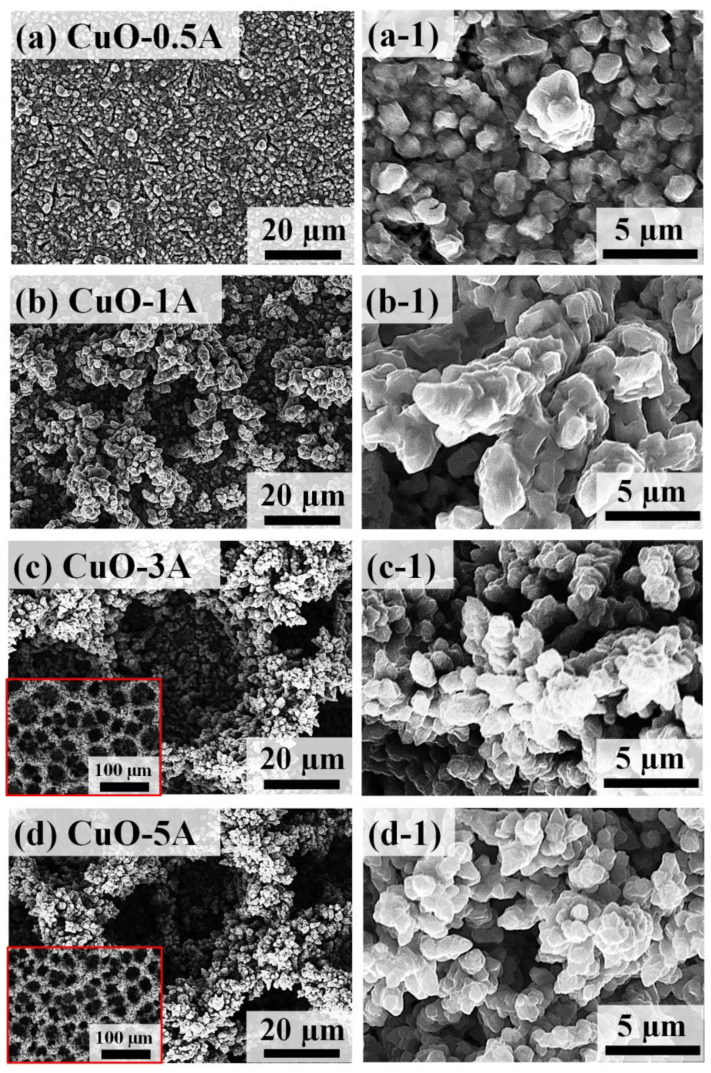
SEM images of porous CuO nanostructures based on the control of current density between 0.5 and 5 A (CuO-0.5A ~ CuO-5A) for the fixed time of 30 s. (**a**–**d**) Large scale SEM images of the CuO nanostructures as labeled. (**a-1**–**d-1**) High-magnification SEM images.

**Figure 6:**
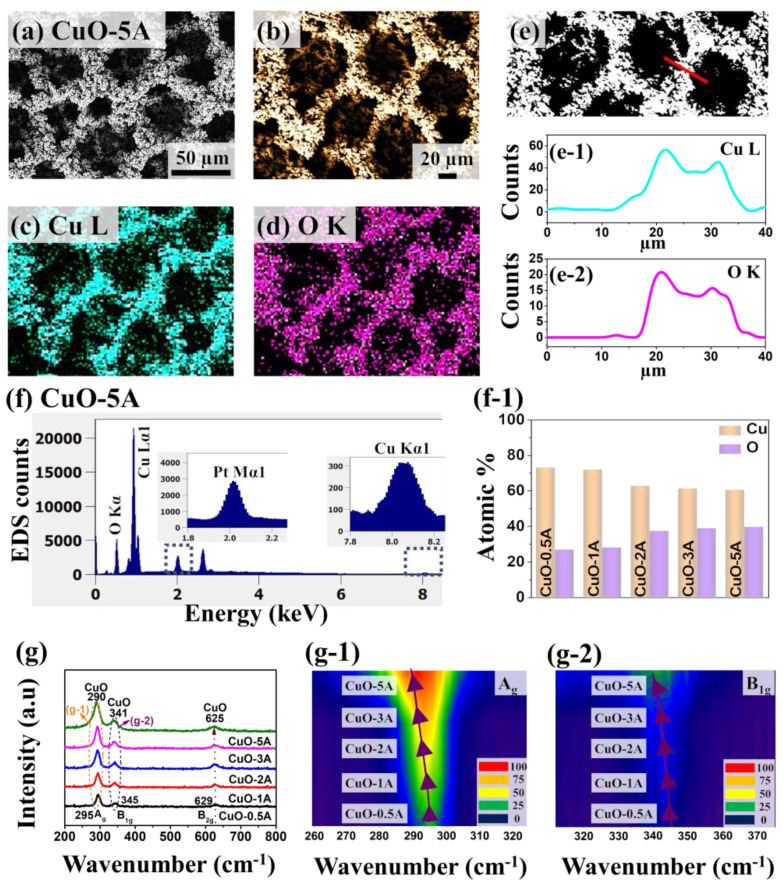
(**a**) SEM image of porous CuO/Pt hybrid detector fabricated at 5 A/cm^2^ for 30 s and denoted as CuO-5A. (**b**–**d**) Enlarged SEM image and elemental maps of Cu and O for the CuO-5A. (**e**–**e-2**) Elemental line-profiles of Cu L and O K. (**f**,**f-1**) EDS spectra of CuO-5A and summary of atomic percentage of Cu and O as a function of deposition current. (**g**) Raman spectra for the porous CuO samples fabricated at different currents. (**g-1**,**g-2**) Contour maps of the Raman peaks of CuO corresponding to the A_g_ and B1_g_ modes.

Figure 5 shows the physical characterization of porous CuO/Pt hybrid electrodes fabricated by the variation of current density between 0.5 and 5 A/cm^2^ on Pt/Si substrate. The SEM images of CuO/Pt hybrid electrodes at different current densities are shown in Figure 5. At a low current density of 0.5 and 1 A/cm^2^, the discrete and irregular vertical growth of dendrite-like structures can be observed on the surface in Figure 5a,b. As the current density was increased further, more porous structures and large vertical dendrites were formed due to the abundant hydrogen bubble formation and clustering of electrodeposited metal atoms in Figure 5c,d. The morphology of porous structures directly depends upon the current density such that higher density and small pore size were obtained with higher current density, which can be due to the faster generation and desorption of hydrogen bubbles [36]. The CV and CA electrochemical response of these various CuO/Pt hybrid samples based on current density variation are presented in Figure S6. Specifically, the CuO-5 A demonstrated the C_dl_ of 0.1005 mF as seen in Figure S6d, which is a higher value than the CuO-30 s as discussed in Figure 3d. The increased C_dl_ further confirms that the CuO-5A sample effectively enhances the catalytic active sites with the increased electrochemical active surface area (ECSA) of the electrode and improves the electron transfer rate for the catalytic performance and demonstrates high sensitivity for H_2_O_2_ reduction. From the CV and CA measurements, the electrochemical performance of CuO-5A for the H_2_O_2_ detection was found to be maximum in this set. Thus, the CuO-5A sample was further explored in terms of physical properties and electrochemical performance for the H_2_O_2_ detection. Figure 6a–d show the SEM and EDS elemental maps of the CuO-5A respectively. The EDS elemental maps demonstrate the presence of Cu L and O K peaks in the CuO-5A and matches well with the SEM morphology. This clearly shows the co-existence of Cu and O elements in the porous nanostructures. In addition, the corresponding EDS line-profile analysis of the selected area is shown in Figure 6e–e-2, which confirms the uniform distribution of Cu and O in the nanostructures. The EDS spectrum of the porous CuO-5A sample in Figure 6f also confirms the presence of Cu, O and Pt elements in the sample. From the atomic percentage plot in Figure 6f-1, the atomic percentage of Cu was gradually reduced while the atomic percentage of O was increased with the increased current density. This indicates that the amount of oxidized Cu is greater in the high current density samples likely due to the higher porosity and pore density. The EDS elemental spectra of other samples in this set are provided in Figure S7. To gain structural insights, Raman spectra analysis was performed as shown in Figure 6g. Generally, the Raman vibration peaks were observed at 295, 345 and 629 cm^−1^ for all CuO samples, which corresponds to A_g_, B_1g_ and B_2g_. In comparison to the Raman peak of the single crystal of CuO, the Raman peaks showed a gradual blue shift with a broadening as shown in Figure 6g-1,g-2. Again, this can be related to the increased grain size along with the increased current [26,37,38]. 

**Figure 7:**
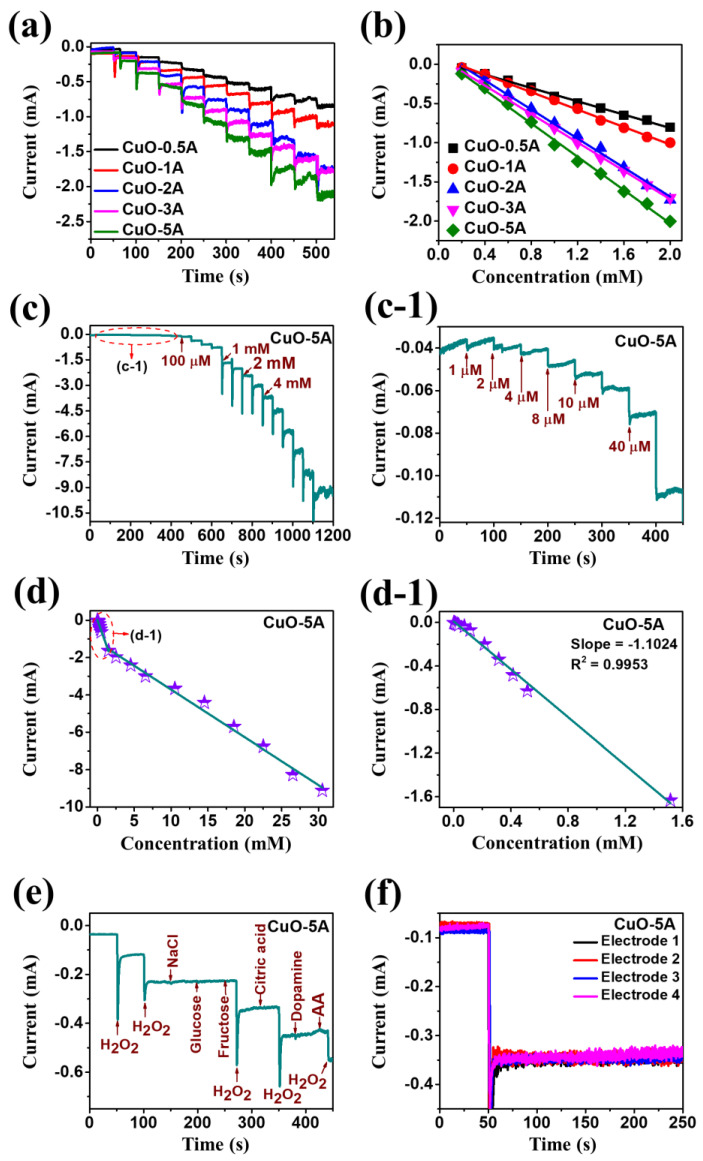
(**a**) Steady-state relation between current and time of current variation set upon successive addition of 0.2 mM H_2_O_2_ in the N_2_-saturated 0.1 M PBS (pH 7.4) at an applied potential of −0.4 V. (**b**) The corresponding calibration curves for H_2_O_2_ detection. (**c**,**c-1**) Amperometric response of CuO-5A upon dropwise addition of various H_2_O_2_ concentrations from 1 µM to 4 mM. (**d**,**d-1**) Calibration curve of current versus H_2_O_2_ concentration at different H_2_O_2_ concentration range. (**e**) Selectivity response of CuO-5A sample upon addition of 0.1 mM H_2_O_2_, NaCl, glucose, fructose, citric acid, dopamine and ascorbic acid (AA) to 0.1 M PBS (pH 7.4). (**f**) Reproductivity test of CuO-5A samples 1−4 in the 0.1 M PBS (pH 7.4) at the potential of −0.4 V.

Figure 7 shows the CA response of porous CuO/Pt hybrid electrodes fabricated at the controlled current density. The CA response was measured by the dropwise addition of H_2_O_2_ concentrations in the N_2_-saturated 0.1 M PBS (pH 7.4) under the stirring condition at a potential of −0.4 V. Figure 7a shows the steady-state amperometric current response of samples upon the dropwise addition of 0.2 mM H_2_O_2_. Specifically, the CuO-5A showed the highest current under the same concentration of H_2_O_2_, indicating the superior diffusion of analyte due to the high porosity and crystallinity of CuO nanostructures. Figure 7b presents the current versus concentration relationship, in which the linear current response was observed as a function of concentration for all the samples and the CuO-5A exhibited the highest current slope. Based on the high current response of the CuO-5A sample, this was further examined for the electrochemical H_2_O_2_ detection. The amperometric response of CuO-5A upon the dropwise addition of H_2_O_2_ from 1 µM to 4 mM is presented in Figure 7c, which clearly showed the current increment upon the addition of H_2_O_2_. The low concentration H_2_O_2_ detection between 1 and 40 µM was further plotted as shown in Figure 7c-1, which presents the steady and stable stepwise current state after the addition of H_2_O_2_. Furthermore, Figure 7d shows the linear calibration curve of current versus H_2_O_2_ concentration ranging from 1 µM to 4 mM. Figure 7d-1 displays the corresponding linear curve of current versus H_2_O_2_ concentration, and the linear equation is given by Equation (4), *y* = −1.1024*x* − 0.339 (R^2^ = 0.9953) for the first linear range, and the sensitivity is calculated to be about 2205 µA/mM·cm^2^ and LOD is 140 nM [35]. In addition, Table 1 summarizes the performance parameters of previously reported CuO-based devices and our biosensors. Our device showed relatively good performance factors compared to other devices in terms of the sensitivity and LOD with a quite wide linear range. Figure 7e shows the selectivity characterization of the CuO-5A sample by varying the various organic molecules of 0.1 mM concentration, i.e., NaCl, glucose, fructose, citric acid, dopamine and ascorbic acid (AA). Interestingly, no current response was observed with the addition of other organic molecules than H_2_O_2_. The alternated molecules dropping sequence showed that the current was sharply increased only for the addition of H_2_O_2_. This result clearly confirms that the CuO-5A is highly selective for the H_2_O_2_ detection at −0.4 V applied bias against typical organic molecules. One step ahead, we measured the selectivity response for the mixture of organic molecules with and without the addition of H_2_O_2_ as shown in Figure S8. Figure S8a,b clearly indicate the sensor very selectively responds to H_2_O_2_ the reduction. The CA response was sharply changed with each drop of 0.1 mM solution with the H_2_O_2_ in Figure S8a. Meanwhile, there was no response for the mixture solution with the H_2_O_2_ in Figure S8b. Thus, it clearly shown that our fabricated biosensor is highly selective for the H_2_O_2_ at −0.4 V. Furthermore, the stability and reproductivity tests were conducted with the working electrode CuO-5A as shown in Figure 7f. During the 250 s test period, all four CuO-5A electrodes exhibited similar and stable current upon the injection of the same concentration of H_2_O_2_. Thus, the porous CuO/Pt hybrid electrodes can be a promising platform for the fabrication of H_2_O_2_ detectors with the high sensitivity, selectivity and reproductivity. This can be attributed to the effective absorption of the H_2_O_2_ on the super-porous CuO interface and fast charge transport pathways through the underlying Pt NPs during the electrolysis process [39].

**Figure 8:**
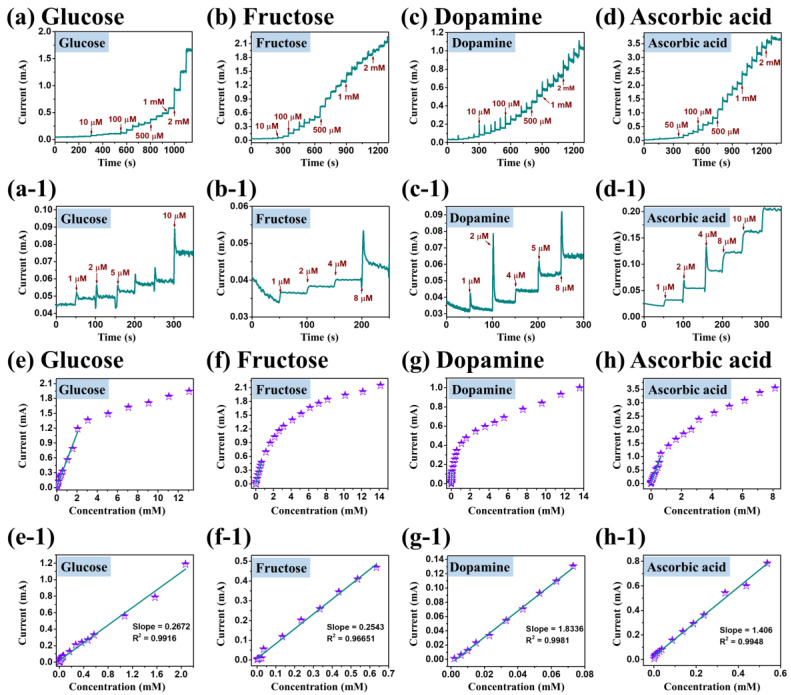
Amperometric response of CuO-5A sample upon dropwise addition of (**a**) glucose into 0.1 M NaOH at a potential of 0.6 V, (**b**) fructose into 0.1 M NaOH at a potential of 0.4 V, (**c**) dopamine into 0.1 M PBS at a potential of 0.6 V and (**d**) ascorbic acid into 0.1 M NaOH at a potential of 0.5 V, respectively. (**a-1**–**d-1**) Amperometric response of glucose, fructose, dopamine and ascorbic acid at lower concentrations. (**e**–**h**) Calibration curves CuO-5A based on the current versus high concentration of glucose, fructose, dopamine and ascorbic acid. (**e-1**–**h-1**) Linear calibration curve based on the current versus low concentration of glucose, fructose, dopamine and ascorbic acid.

Figure 8 presents the typical amperometric responses of CuO-5A for the detection of different organic molecules at different measurement conditions such as (a) glucose in 0.1 M NaOH at 0.6 V (b) fructose in 0.1 M NaOH at 0.4 V (c) dopamine in 0.1 M PBS at 0.6 V and (d) ascorbic acid in 0.1 M NaOH at 0.5 V. All the measurements were carried out the under a N_2_ saturation and stirring condition. Since, the applied potential has a great impact on the sensitivity, stability and selectivity of sensors, the applied potential for different molecules was determined based on the CV and CA measurement as shown in Figure S9 [48]. Figure 8a–d show the amperometric response of the CuO-5A sample upon the dropwise addition of different molecules as labeled with various concentrations ranging from 1 µM to 2 mM. Figure 8a-1–d-1 present the magnified section of Figure 8a–d at low concentration ranging from 1–10 µM. It was found that the CuO-5A sample exhibited a decent current response with the consequent step change for each drop of different molecules at different potentials. These results confirm that the porous CuO/Pt hybrid electrodes can exhibit excellent sensitivity with different organic molecules, which can be ascribed to high electroconductivity and good electrocatalytic activity. The corresponding linear calibration curves of current versus concentration of each molecules at high concentration ranging from 10 µM to 2 mM is shown in Figure 8e–h. Similarly, the low concentration 1–10 µM linear calibration curves are presented in Figure 8e-1–h-1 for different molecules as labeled. From these results, it can be concluded that the porous CuO/Pt hybrid electrodes can be applied for the detection of various organic molecules at different applied potentials as well.

## 4. Conclusions

In summary, a significantly improved H_2_O_2_ detection performance has been demonstrated by the uniquely designed super-porous hybrid nanostructures of CuO/Pt on Si fabricated by the combined physicochemical approach. In particular, the physical vapor deposition of Pt NPs on the Si substrate was followed by the electrochemical deposition of highly porous nanostructures of CuO by the dynamic hydrogen bubbles approach at different deposition times and current densities. It has been found that the performance of the H_2_O_2_ sensor is highly dependent on deposition conditions. The highly porous structure of CuO deposited at a current density of 5 A/cm^2^ for 30 s (CuO-5A) showed the best performance with the highest sensitivity, wide linear range and selectivity towards the H_2_O_2_. Under the optimized conditions, the CuO-5A demonstrated the sensitivity of 2205 µA/mM·cm^2^ with wide detection range. It also demonstrated an excellent selectivity against other organic molecules like glucose, fructose, dopamine and ascorbic acid along with the limit of detection of 140 nM. The enhanced sensing performance was due to the increased active sites and improved H_2_O_2_ adsorption and interfacial electron transport, which was achieved by a unique manufacturing method of the dynamic hydrogen bubble technique. Furthermore, the CuO-5A showed the detection of other organic materials at different applied potential. This work demonstrates that the highly porous CuO/Pt platform could be a promising candidate to develop an efficient electrochemical biosensor for the H_2_O_2_ sensor application.

## Figures and Tables

**Table 1:** Comparison of electrochemical H_2_O_2_ sensing performance for the CuO-based materials.

Electrode Material	Limit of Detection	Linear Range	Electrolyte Solution	Sensitivity [µA mM^−1^ cm^−2^]	Reference
CuO-5A	140 nM	1 µm–1.5 mM	0.1 M PBS	2205	**Present** **Work**
CuO-30 s	325 nM	1 µm–1.5 mM	0.1 M PBS	1498	
NP-PdCu	1.9 µM	0.1–30 mM	PBS + 1 mM H_2_O_2_	1.6	[9]
Cu_2_O PLNWs/Cu foam	1.05 µM	5–1770 µM	0.1 M NaOH	1.4773	[11]
CuO-NP	1.6 µM	0.01–13.18 mM	0.1 M PBS	22.27	[19]
CuO nanorods	-	0.25–18.75 mM	0.1 M NaOH	84.89	[40]
CuO nanosheet	10 µM	10–20,000 µM	1 M NaOH	25.5	[18]
CuO@Cu_2_O-NWs/PVA/GC	0.35 µM	1 µM–3 mM	0.1 M NaOH	39.5	[41]
CuO/rGO/Cu_2_O	0.05 µM	0.5 µM–9.7 mM	0.1 M NaOH	366.2	[42]
ZnO_3_-CuO_7_/CPE	2.4 µM	0.003–0.53 mM	0.1 M KCl	1.11	[43]
CuOx/NiOy	90 nM	0.03 µM–9.0 mM	0.10 M NaOH	271.1	[44]
3D CuO/Cu	2 µM	2 µM–19.4 mM	0.1 M NaOH	103	[45]
CuO nanostructures	43 nM	250 nm–2 mM	0.1 M PB	2015.7	[46]
3 DOI Au/NiO@CuO	3.7 nM	20 nM–20 µM	0.1 M PBS	650.2	[47]

## Supplementary Materials

The following are available online at https://www.mdpi.com/2079-4991/10/10/2034/s1, Figure S1. (a–c) Atomic force microscope (AFM) image of Si substrate before degassing, after degassing and after 50 nm Pt layer deposition and annealing at 425 °C for 30 min. (a-1–c-1) Cross-sectional line profiles from the corresponding AFM images. (d) Summary of RMS roughness (Rq) and surface area ratio (SAR) under different conditions. (d-1) Rq and SAR summary table, Figure S2. (a–d) Schematics of porous CuO fabrication procedure on Pt/Si, Figure S3. (a–e) Large scale SEM images of porous CuO samples by the electrochemical deposition time variation at 2 A/cm^2^ cathodic current density. (a-1–e-1) High magnification SEM images of the porous CuO, Figure S4. EDS spectra of porous CuO based on the variation of deposition time (a) CuO-5 s, (b) CuO-10 s, (c) CuO-20 s, and (d) CuO-50 s. Insets show the enlarged Pt Mα1 and Cu Kα1 peaks in each sample. The elemental composition of Cu and O is summarized in corresponding tables, Figure S5. (a,b) CV responses of Si substrate and Pt/Si substrate with and with the addition of the H_2_O_2_ in 0.1 mM PBS (pH 7.4) at a scan rate of 50 mV/s. (c) Amperometric response of Si substrate and Pt/Si substrate and (d) linear calibration curve of current versus concentration of H_2_O_2_ with Pt/Si substrate, Figure S6. (a) Cyclic voltammetry (CV) response of various CuO samples in 0.1 M PBS (pH 7.4) containing 0.4 mM H_2_O_2_ at a scan rate of 50 mV/s. (b) Amperometric response of CuO-5A sample with dropwise addition of 0.1 mM H_2_O_2_ at different applied potential. (c) CVs response of the CuO-5A sample at different scan rates from 20 to 200 mV/s in 0.1 M PBS (pH 7.4) containing 0.1 mM H_2_O_2_. (d) Corresponding capacitive plot current Vs square root of scan rate for CuO-5A at −0.2 V (*Δj*_−0.2_ = (*j_a_* − *j**_c_***)/2). (c,e) CV of CuO-5A sample in 0.1 M PBS (pH 7.4) containing different concentrations of H_2_O_2_ ranging from 0.5 to 3.5 mM at the scan rate of 50 mV/s. (f) Relation between peak current and H_2_O_2_ concentration at −0.2 V. Figure S7. (a–d) EDS spectra of CuO samples at different deposition current density CuO-0.5A–CuO-3A. Insets show the enlarged Pt Mα1 and Cu Kα1 peaks. The elemental composition of Cu and O is summarized in corresponding tables, Figure S8. Amperometric response of CuO-5A sample upon the successive addition of mixture solution of 0.1 mM dopamine (DA), ascorbic acid (AA) and and Uric acid (UA) (a) with H_2_O_2_ and (b) without H_2_O_2_ to 0.1 M PBS (pH 7.4) at applied potential of −0.4 V. Figure S9. (a–g) CV response of CuO-5A with and without the addition of 0.1 mM organic molecules such as glucose, fructose, and ascorbic acid in 0.1 M NaOH and, dopamine in 0.1 M PBS at a scan rate of 50 mV/s. (b–d) Amperometric current response of corresponding organic molecules at different applied potentials.
