# Supplementary material for: Hydrogen Peroxide Detection by Super-Porous Hybrid CuO/Pt NP Platform: Improved Sensitivity and Selectivity
Source: Nanomaterials (Basel). 2020 Oct 15;10(10):2034. doi: 10.3390/nano10102034 (PMC7602549; doi:10.3390/nano10102034)
Supplement: Supplementary file 1 [file nanomaterials-10-02034-s001.pdf]

Supplementary Information

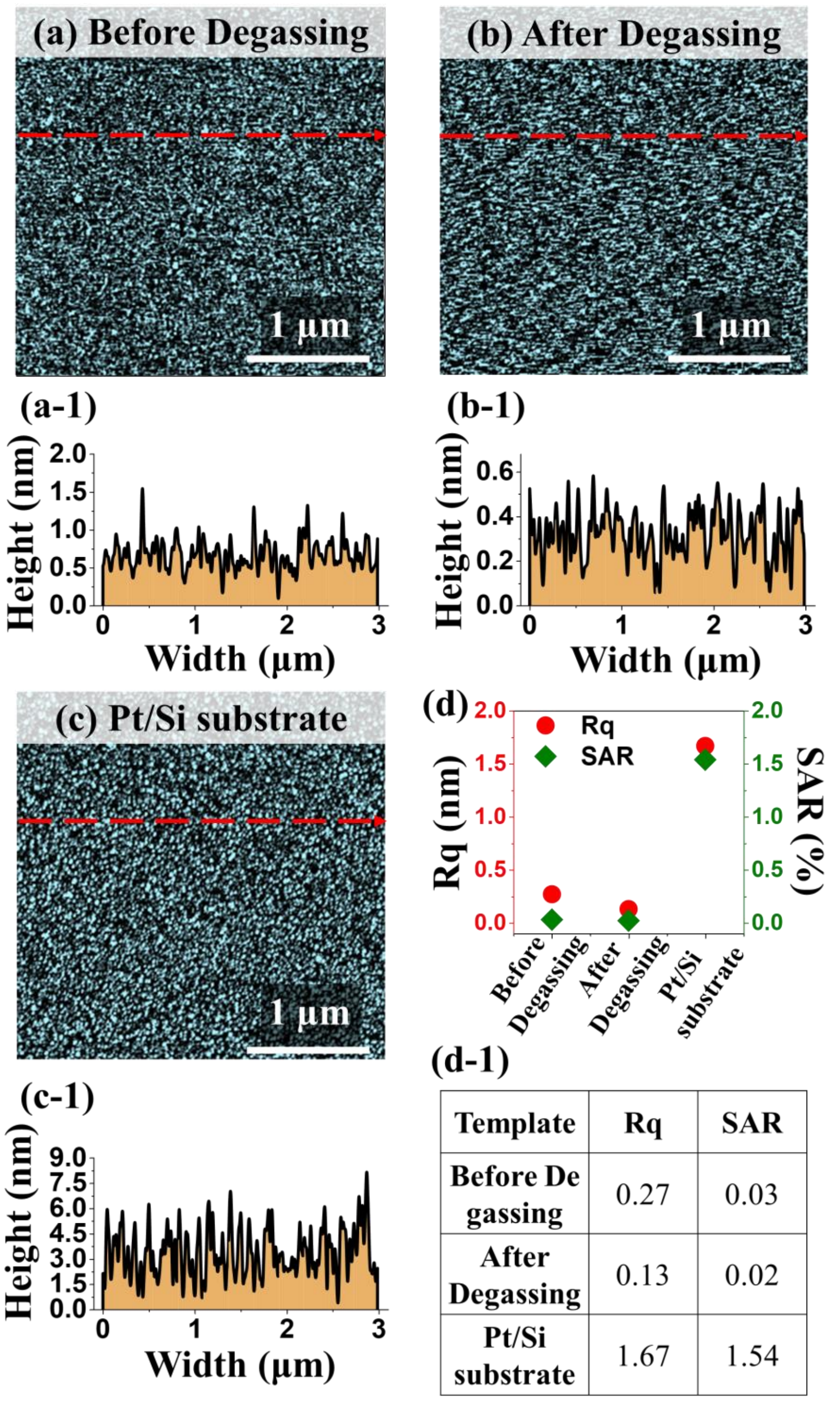

Template	Rq	SAR
Before De gassing	0.27	0.03
After Degassing	0.13	0.02
Pt/Si substrate	1.67	1.54

Figure S1. (a)-(c) Atomic force microscope (AFM) image of Si substrate before degassing, after degassing and after 50 nm Pt layer deposition and annealing at 425 °C for 30 min. (a-1) – (c-1) Cross-sectional line profiles from the corresponding AFM images. (d) Summary of RMS roughness (Rq) and surface area ratio (SAR) under different conditions. (d-1) Rq and SAR summary table.

.

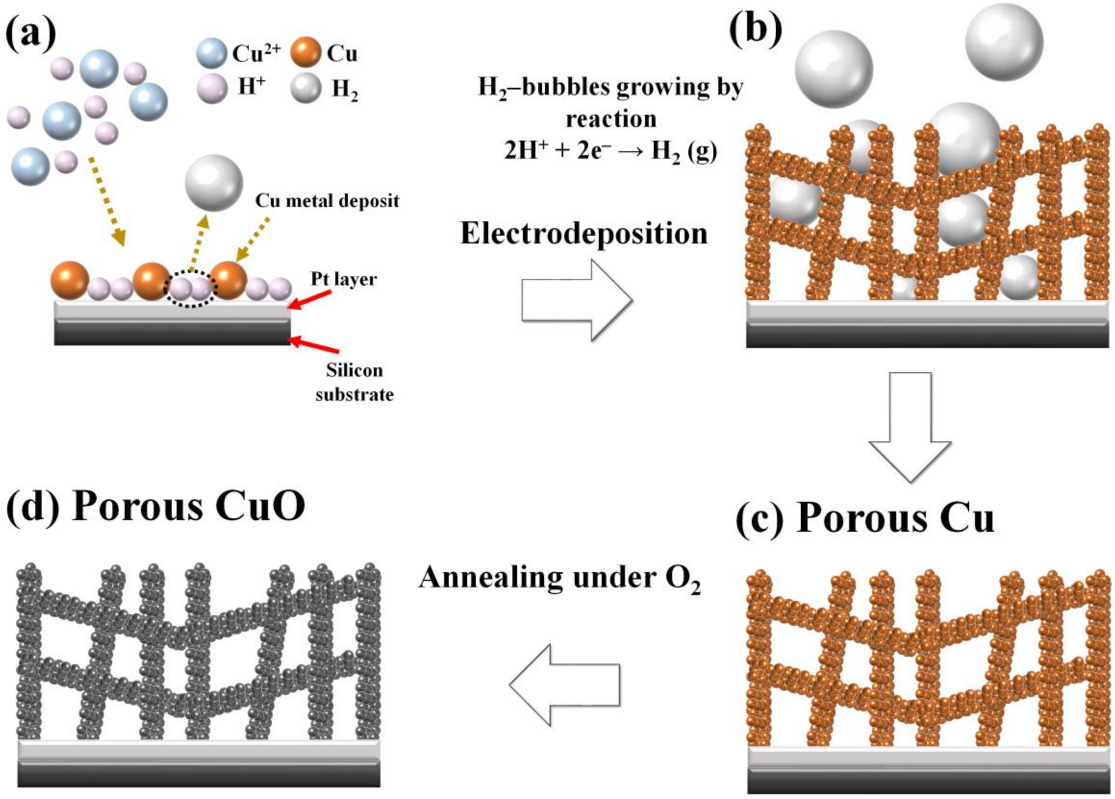

Figure S2. (a) – (d) Schematics of porous CuO fabrication procedure on Pt/Si. As shown in (a), the deposition of Cu ions and the evolution of hydrogen bubbles occurs simultaneously on the surface during electrochemical deposition. (b) As the H_2 bubble generation continues, metal ions grow in between the gas bubbles. These H_2 bubbles can create dynamic templates for the growth of Cu nanostructures. (c) Pores in the range of micron size are generated by the growth of Cu around the bubbles, resulting in the highly porous metallic layer. The agitation action of the bubbles affects the hydrodynamic conditions near the electrode surface and greatly affects the subsequent nanostructure. (d) Various nanostructures such as dendrites, small pores to large pores can be created along with the control of deposition conditions such as current density and deposition time. Due to the large overpotential used, a co-reduction process occurs in which the metal ions in the electrolyte are reduced simultaneously with H^+, i.e., the processes in which equations 1 and 2 occur simultaneously.

$$2\mathrm{H}^{+}{}_{(\mathrm{aq})} + 2\mathrm{e}^{-} \rightarrow \mathrm{H}_2 \ (1)$$

$$Cu^{2+}{}_{(\mathrm{aq})} + 2\mathrm{e}^{-} \rightarrow \mathrm{Cu}_{(\mathrm{s})} \ (2)$$

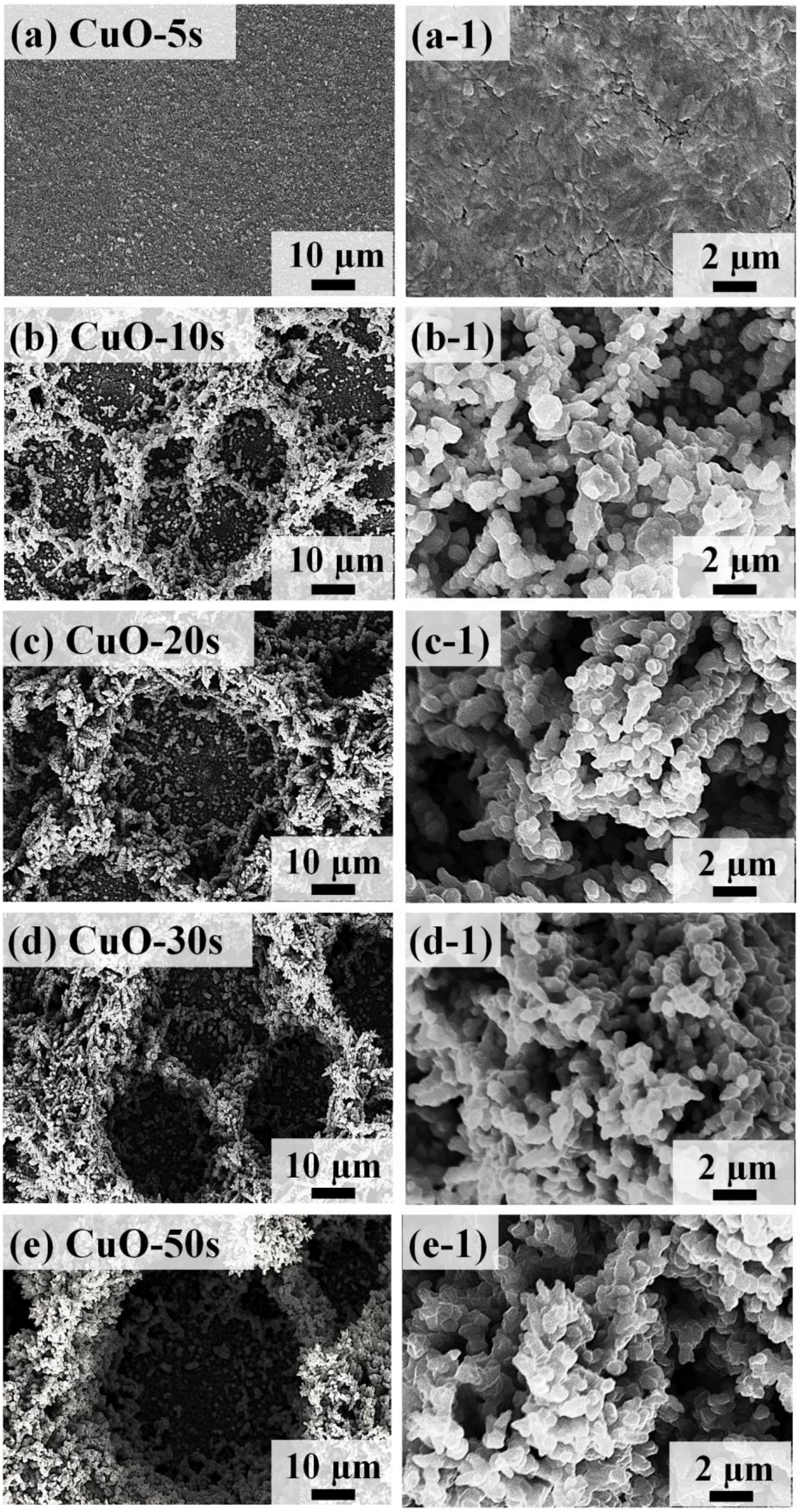

Figure S3. (a) – (e) Large scale SEM images of porous CuO samples by the electrochemical deposition time variation at 2 A/cm^2 cathodic current density. (a-1) – (e-1) High magnification SEM images of the porous CuO.

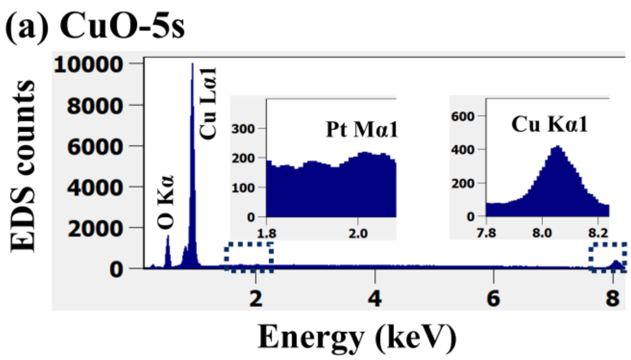

Element	Wt. %	At. %
Cu	91.58	73.26
O	8.42	26.74

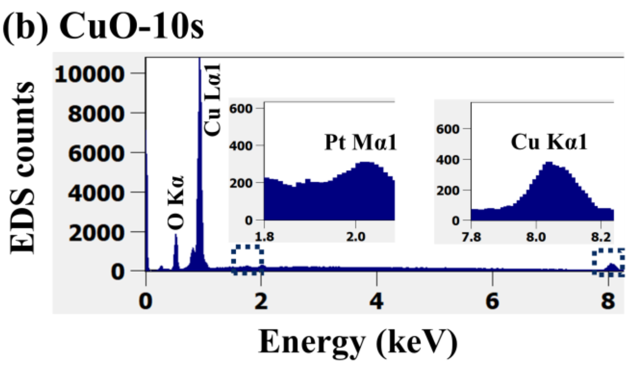

Element	Wt. %	At. %
Cu	90.71	71.08
O	9.29	28.92

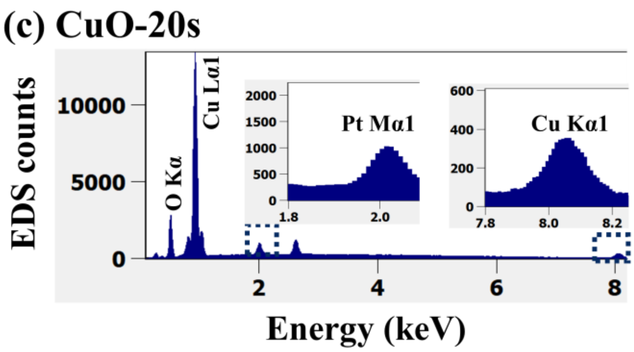

Element	Wt. %	At. %
Cu	88.55	66.08
O	11.45	33.92

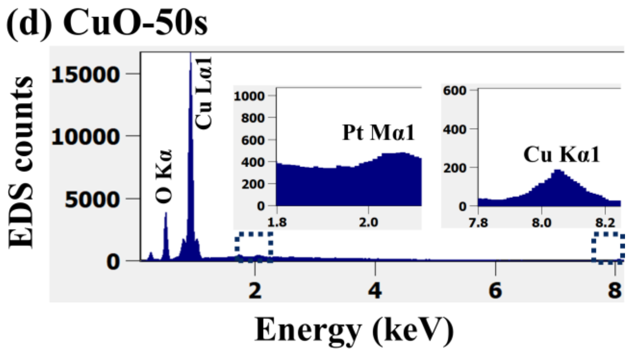

Element	Wt. %	At. %
Cu	87.62	64.05
O	12.38	35.95

Figure S4. EDS spectra of porous CuO based on the variation of deposition time (a) CuO-5 s, (b) CuO-10 s, (c) CuO-20 s, and (d) CuO-50 s. Insets show the enlarged Pt Mα1 and Cu Kα1 peaks in each sample. The elemental composition of Cu and O is summarized in corresponding tables. .

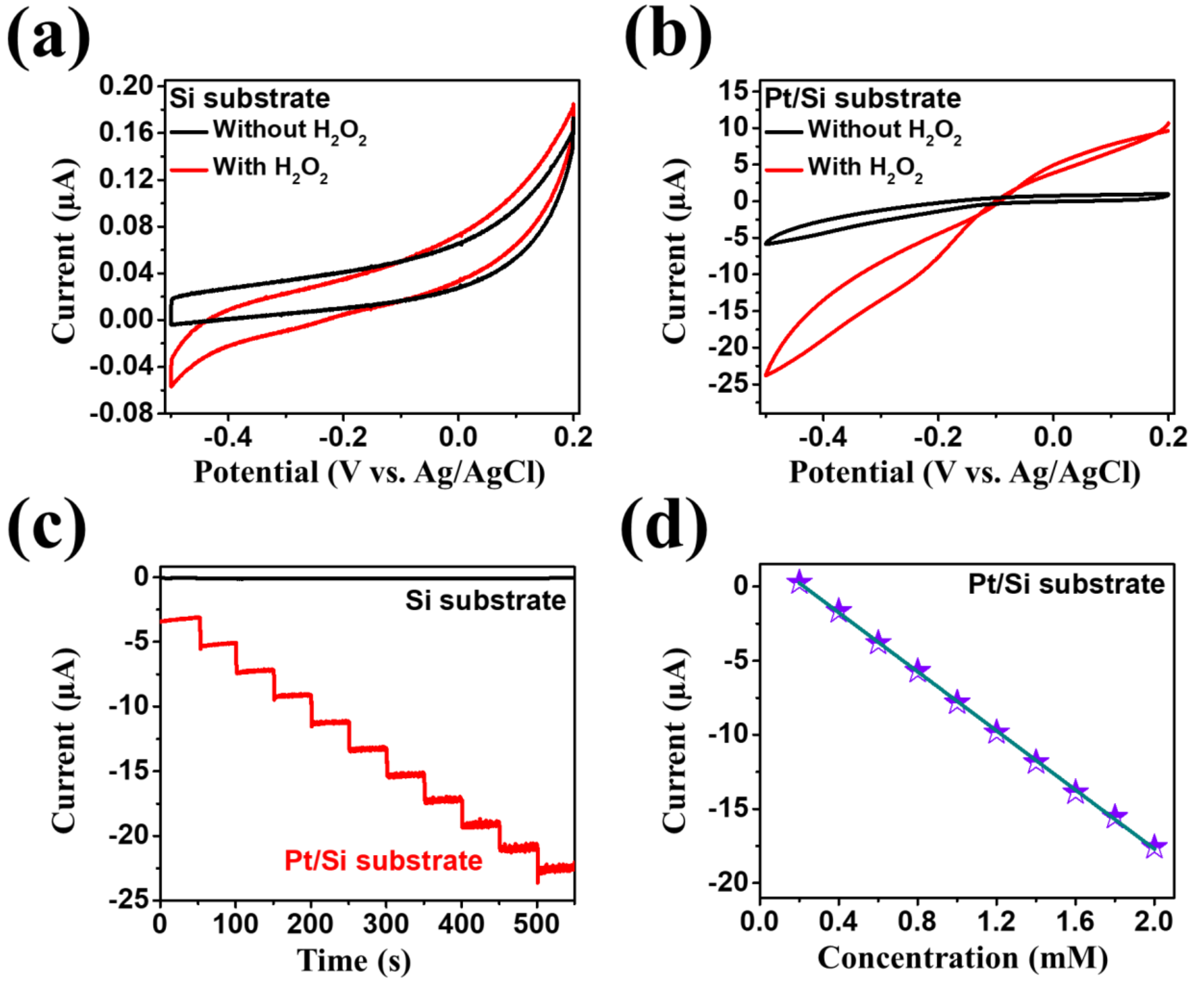

Figure S5. (a) – (b) CV responses of Si substrate and Pt/Si substrate with and with the addition of the H_2O_2 in 0.1 mM PBS (pH 7.4) at a scan rate of 50 mV/s. (c) Amperometric response of Si substrate and Pt/Si substrate and (d) linear calibration curve of current versus concentration of H_2O_2 with Pt/Si substrate.

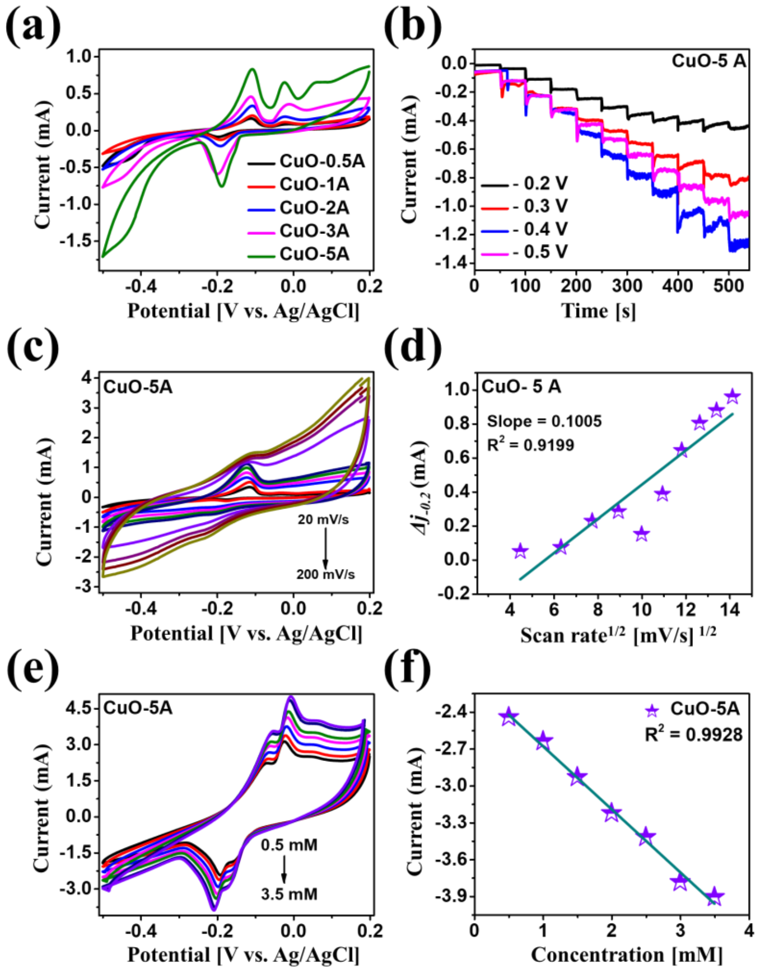

Figure S6. (a) Cyclic voltammetry (CV) response of various CuO samples in 0.1 M PBS (pH 7.4) containing 0.4 mM H_2O_2 at a scan rate of 50 mV/s. (b) Amperometric response of CuO-5A sample with dropwise addition of 0.1 mM H_2O_2 at different applied potential. (c) CVs response of the CuO-5A sample at different scan rates from 20 to 200 mV/s in 0.1 M PBS (pH 7.4) containing 0.1 mM H_2O_2. (d) Corresponding capacitive plot current Vs square root of scan rate for CuO-5A at -0.2 V ($\Delta j_{-0.2} = (j_a - j_c)/2$). (c). (e) CV of CuO-5A sample in 0.1 M PBS (pH- 7.4) containing different concentrations of H_2O_2 ranging from 0.5 to 3.5 mM at the scan rate of 50 mV/s. (f) Relation between peak current and H_2O_2 concentration at -0.2 V.

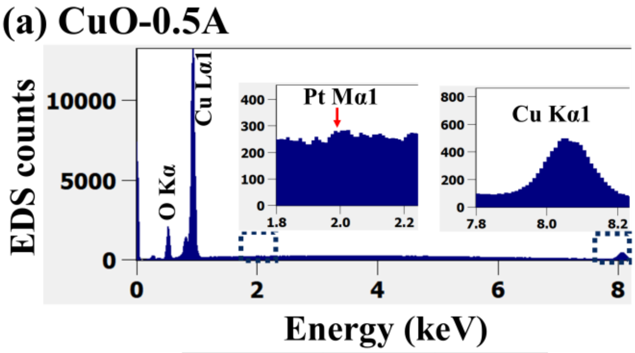

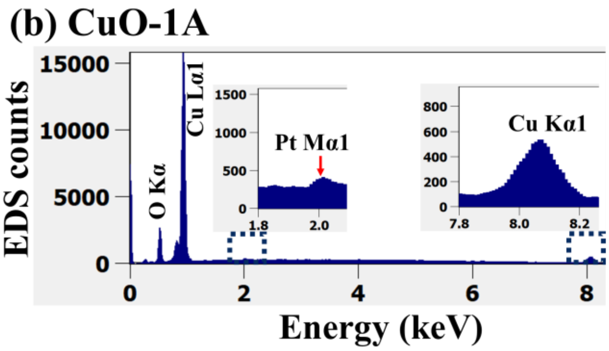

Element	Wt. %	At. %
Cu	91.53	73.11
O	8.47	26.89

Element	Wt. %	At. %
Cu	91.05	71.93
O	8.95	28.07

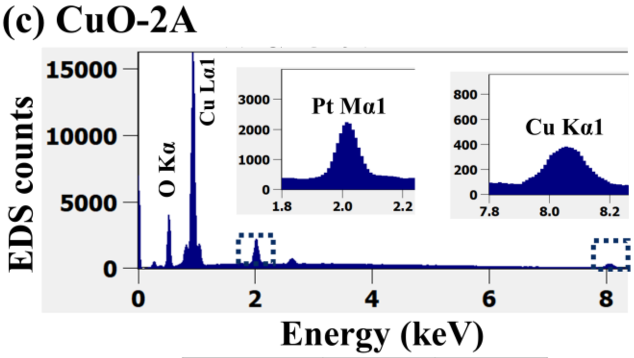

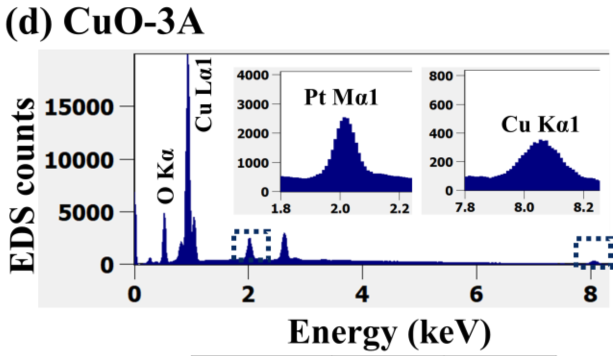

Element	Wt. %	At. %
Cu	86.99	62.73
O	13.01	37.27

Element	Wt. %	At. %
Cu	86.25	61.23
O	13.75	38.77

Figure S7. (a) – (d) EDS spectra of CuO samples at different deposition current density CuO-0.5A - CuO-3A. Insets show the enlarged Pt Mα1 and Cu Kα1 peaks. The elemental composition of Cu and O is summarized in corresponding tables. .

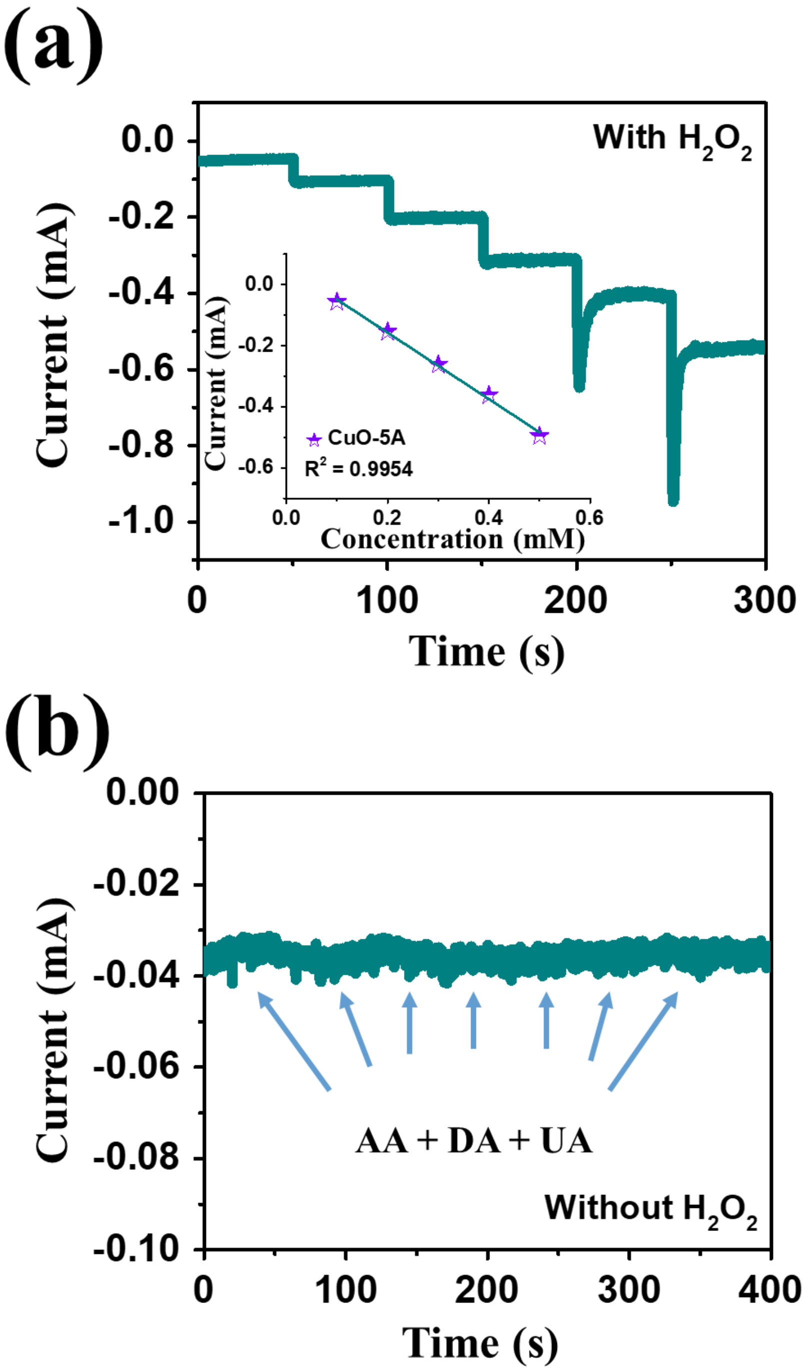

Figure S8. Amperometric response of CuO-5A sample upon the successive addition of mixture solution of 0.1 mM dopamine (DA), ascorbic acid (AA) and and Uric acid (UA) (a) with H_2O_2 and (b) without H_2O_2 to 0.1 M PBS (pH 7.4) at applied potential of - 0.4 V.

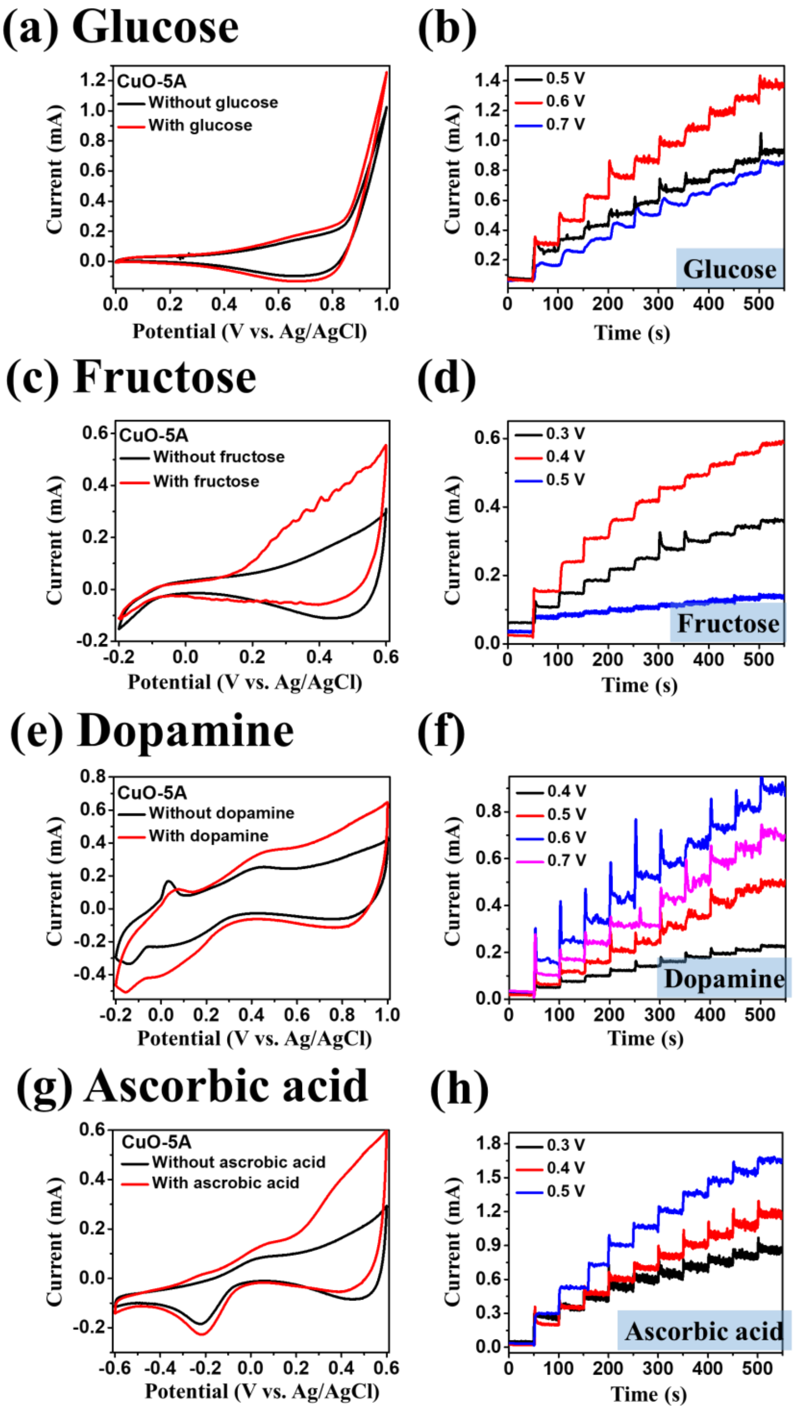

Figure S9. (a) – (g) CV response of CuO-5A with and without the addition of 0.1 mM organic molecules such as glucose, fructose, and ascorbic acid in 0.1 M NaOH and, dopamine in 0.1 M PBS at a scan rate of 50 mV/s. (b) – (d) Amperometric current response of corresponding organic molecules at different applied potentials.
